# sTREM2 cerebrospinal fluid levels are a potential biomarker in amyotrophic lateral sclerosis and associate with UMN burden

**DOI:** 10.3389/fneur.2024.1515252

**Published:** 2024-12-10

**Authors:** Lin Jiao, Jing Yang, Wenjing Wang, Xiangyi Liu, Yu Fu, Dongsheng Fan

**Affiliations:** ^1^Department of Neurology, Peking University Third Hospital, Beijing, China; ^2^Beijing Key Laboratory of Biomarker and Translational Research in Neurodegenerative Diseases, Beijing, China; ^3^Department of Anesthesiology, Peking University Third Hospital, Beijing, China; ^4^Key Laboratory for Neuroscience, National Health Commission/Ministry of Education, Peking University, Beijing, China

**Keywords:** soluble TREM2, cerebrospinal fluid, amyotrophic lateral sclerosis, MBS, UMN burden

## Abstract

**Objectives:**

The aims of this study were to investigate whether CSF sTREM2 may be a potential marker of disease monitoring for amyotrophic lateral sclerosis (ALS).

**Methods:**

We investigated whether CSF sTREM2 levels are altered in ALS patients and are correlated with upper motor neuron (UMN) burden and disease progression.

**Results:**

CSF sTREM2 was greater in the ALS patients than in the controls (*p* = 0.002). Elevated CSF sTREM2 was associated with the UMN score (*r* = 0.38, *p* = 0.009), ΔFS (*r* = 0.30, *p* = 0.04) and serum NFL (lg) (*r* = 0.35, *p* = 0.015). As the motor band sign (MBS) score increased, the CSF sTREM2 level increased (*p*-trend = 0.014). Furthermore, the correlations became stronger (UMN score (*r* = 0.50, *p* = 0.01) ΔFRS (*r* = 0.52, *p* = 0.008) and serum NFL (lg) (*r* = 0.55, *p* = 0.004) when estimated only among patients with a disease duration >12 months.

**Conclusion:**

We found that CSF sTREM2 is elevated in ALS patients and may be a novel marker, probably reflecting upper motor unit severity and prognosis.

## Introduction

ALS is a fatal central nervous system neurodegenerative disease and presents as a combination of upper (UMN) and lower motor neuron (LMN) dysfunction that leads to progressive weakness of voluntary skeletal muscles involved in limb movement (1). The challenge for current research is to discover novel ALS biomarkers to identify or monitor disease progression (2). In particular, LMN burden can be quantified by electromyography, but few reliable biomarkers are available to quantify UMN impairment.

Research on postmortem tissue and animal models has revealed an association between microglia and ALS (3, 4). The intricate cellular interplay between immune cells and upper motor neurons has been observed in the motor cortex of both ALS mice and ALS patients (5). Axonal loss in the corticospinal tract is associated with microglia (6). Therefore, the activation of microglia could be an attractive biomarker for tracking this disease and a potential outcome parameter for future clinical trials.

However, it is difficult to directly observe central microglia in clinical practice. Recently, CSF soluble TREM2 (sTREM2) has been evaluated as a biomarker of microglial activation in the context of neuroinflammatory and neurodegenerative diseases (7). Since sTREM2 is a key protein involved in the activation of microglia, the question arises whether sTREM2 levels are pathologically altered in the ALS.

In this study, we first compared CSF sTREM2 protein levels in patients with ALS and controls. We then evaluated the association of sTREM2 with clinical presentation. We found that CSF sTREM2, which may be a candidate marker for ALS disease monitoring, is significantly elevated in ALS patients and is associated with UMN burden and disease progression.

## Materials and methods

### Study participants

The current study included 47 sporadic ALS patients [according with the El Escorial revised criteria (8)] and 23 controls from the Peking University Third Hospital (PUTH). We included as CSF-controls subjects who underwent an intraspinal anesthesia in minor surgery and match for age and sex with ALS group. All participants met the following features: no other neurodegenerative disease such as Alzheimer’s disease, no tumoral or systemic inflammatory disease and no active anti-inflammatory drugs intake. The study was approved by the ethics committee of PUTH (M2024097).

For all patients, baseline demographic information and clinical data were collected directly during the patient’s first visit to PUTH. The demographic and clinical characteristics included age, sex, site of onset, age at onset, disease duration, and disease extent as assessed by the ALS Functional Rating Score-Revised (ALSFRS-R).

### Indicators of UMN burden

#### UMN score

UMN score was assessed by Penn’s upper motor neuron scale (9).

#### Motor band sign

Research with Quantitative Susceptibility Mapping (QSM) proposed MBS can serve as a possible radiological sign of severe UMN burden in ALS patients (10). Nineteen ALS participants agreed to undergo a full brain MRI protocol, including T2*/GRE sequence. MRI was performed using 3-T clinical magnetic resonance system (MAGNETOM TRIO TIM, Siemens, Germany). T2*/GRE sequence scans were performed using the T2*-weighted gradient echo protocol. 20-channel head-and-neck collar was used. Imaging sequence parameters were as follows: repetition time 800 ms, echo time 30 ms, flip angle 20°, slice thickness 5 mm, phase FOV 128 × 128 mm. All SWI sequences were assessed on radiological PACS workstations (GE) independently by 2 doctors. Raters were blinded to the participant’s age, sex and other clinical information. The raters evaluated the degree of hypointensity in the motor cortex on SWI using a three-point ordinal scale from 0 to 2, as previously reported (11, 12): a total MBS score of 0–12: The human primary motor cortex is somatotopically organized into 3 regions, the most medial part, the most lateral parts and the intermediate region. Compared with the peripheral gyrus, each region obtains a score, which is isointense (0), mildly hypointense (1), and markedly hypointense (2).

### Indicators of disease progression

#### ΔFS

The progression rate (ΔFS) was calculated as follows: ΔFS (13) = (48 − ALSFRS-R Score at Time of Diagnosis)/Duration From Onset to Diagnosis (Months).

#### Serum NFL measurements

As a marker of axonal injury, serum NFL has been shown to be associated with progression and prognosis in patients with ALS (14). Serum NfL levels were measured with an ultrasensitive single-molecule array (Simoa) platform provided by Quanterix (Lexington, MA, United States). Measurements were performed on the fully automated HD-1 Analyzer (Quanterix) instrument using the NF-L Beta kit from Quanterix, which employs an anti-NFL monoclonal antibody produced by UmanDiagnostics (Umeå, Sweden). The interassay coefficients of variation (CVs) were <10%.

#### CSF sTREM2 measurements

The CSF samples were collected in polypropylene tubes immediately by standard lumbar puncture and sent to the laboratory in 2 h. Samples were centrifuged at 2,000 × g for 10 min, and then stored in enzyme-free EP tubes at −80°C. The thaw/freezing cycle was limited not to surpass 2 times. CSF sTREM2 was determined with one ELISA kit (Human TREM2 SimpleStep ELISA kit; Abcam, No. Ab224881). Measurements were performed on the microplate reader (Tecan Spark, Switzerland). The within-batch CV was <5% and the inter-batch CV was <20%.

Cerebrospinal fluid and serum samples were collected on the same day. The measurements were performed in duplicate by experienced technicians who were blind to the clinical information, and the mean concentrations of duplicates were selected for the statistical analyses.

### Statistical data analysis

The Shapiro–Wilk test (S–W test) was used to evaluate whether continuous variables conformed to a normal distribution. Quantitative variables are expressed as the means ± standard deviations if they follow a normal distribution; otherwise, they are expressed as medians (interquartile ranges). Qualitative variables are expressed as a percentage of each group. The expression levels of sTREM2 in cerebrospinal fluid and NFL in serum were converted to log 10(X) values to conform to normal conditions.

Differences in baseline features were determined via the *χ*^2^ test (for categorical variables) and the Mann–Whitney *U* test (for continuous variables).

With sex and age as covariables, the independent correlation between the CSF sTREM2 level and ALS status was evaluated via a logistic regression model, and the odds ratio (OR) was calculated.

Cerebrospinal fluid sTREM2 levels were correlated with clinical data via Pearson and Spearman correlations.

The *χ*^2^ trend test was used to examine the trend of sTREM2 (dividing sTREM2 into 2 groups on the basis of the median) to change with MBS score.

Two-sided *p*-values <0.05 were considered statistically significant.

SPSS 26.0 was used for statistical analysis. Graphs were generated via GraphPad Prism 9.5.

## Results

### Increase of CSF sTREM2 levels in ALS

**Figure 1:**
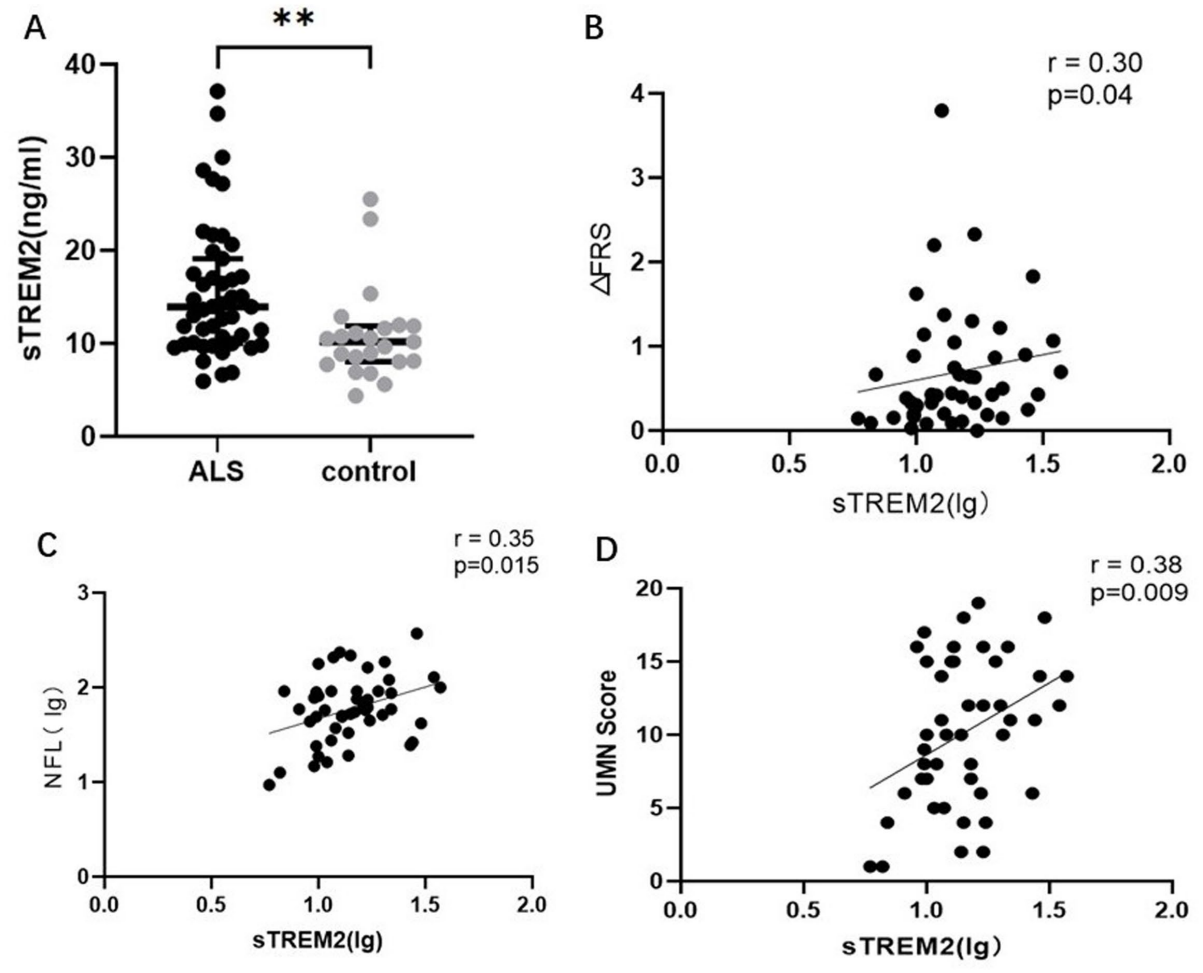
**(A)** The levels of soluble sTREM2 are significantly greater in ALS patients than in controls (*p* < 0.05). **(B)** Positive correlation between sTREM2 levels (lg) and the ΔFRS. **(C)** The positive correlation between sTREM2 levels (lg) and NFL. **(D)** Positive correlation between sTREM2 levels (lg) and the UMN score.

In this study, we aimed to verify whether sTREM2 levels are associated with ALS. We performed intergroup comparisons of participant characteristics and CSF sTREM2 levels. The demographic and biomarker values of the control and ALS patients are shown in Table 1. Age and gender did not differ between the groups. As expected, the groups differed with respect to their biomarker profiles (Figure 1). Logistic regression models were designed to identify adjusted estimates of the association of sTREM2 levels in CSF with ALS status (control = 0; ALS = 1). sTREM2 levels in CSF were an independent predictor of ALS status after adjusting for age and sex, with an odds ratio (OR) = 1.22 (CI 95% = 1.06–1.40, *p* = 0.005). Compared with those in controls, the levels of CSF sTREM2 in ALS patients are significantly greater and may be an independent risk factor for ALS.

**Table 1:** Demographic and clinical characteristics of the control and ALS groups.

	ALS (*N* = 47)	Control (*N* = 23)	*p*
Age	55.40 ± 9.69	56.00 ± 9.60	>0.05
Sex (male/female)	24/23	13/10	>0.05
Disease course (months)	14 (13)	—	—
Site of onset (bulbar/spinal)	10/37	—	—
ALS-FRS	41 (8)	—	—
ΔFS	0.43 (0.70)	—	—
KCSS (1/2/3/4)	21/19/5/2	—	—
UMN score	10.15 ± 4.92	—	—
Biomarkers			
sTREM2 (ng/mL)	13.94 (9.08)	10.20 (3.84)	0.002
lg sTREM2	1.15 ± 0.19	1.00 ± 0.17	0.002
NFL (pg/mL)	58.62 (53.74)	—	—
lg NFL	1.77 ± 0.39	—	—

### CSF sTREM2 is associated with UMN burden

**Figure 2:**
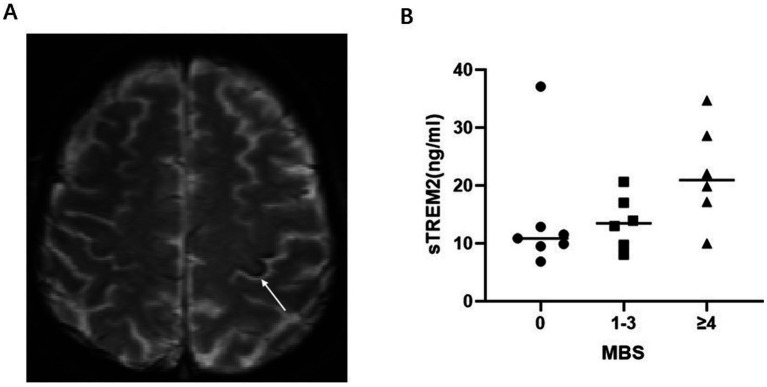
**(A)** An ALS patient with T2* intermediate regions of the motor cortex with marked hypointensity. **(B)** CSF sTREM2 at different MBS levels.

Next, considering the close association between microglia in the central nervous system and motor units in the cortex, we explored whether there was an association between sTREM2 and UMN burden. We collected and analyzed the UMN score and MBS, as previously described. The UMN score was significantly positively correlated with the sTREM2 level (*r* = 0.38, *p* = 0.009) (Figure 1). In addition, we compared sTREM2 levels across different MBS score groups. Adjusting for changes in the MBS, the levels of sTREM2 increased (*p*-trend = 0.014) (Figure 2). These findings suggest that sTREM2 may reflect the potential effects of activated microglia on nearby motor neurons.

### CSF sTREM2 is associated with axonal injury and disease progression

We studied the relationships between CSF sTREM2 and biomarkers of axonal injury and disease progression using Pearson and Spearman correlations. A significant correlation was found between the sTREM2 level and the serum NFL level (*r* = 0.35, *p* = 0.015) and between the sTREM2 level and the ΔFS value (*r* = 0.30, *p* = 0.04) in ALS patients (Figure 1). Interestingly, we found no correlation between CSF sTREM2 levels and the ALSFRS-R score (*p* = 0.15) or disease course (*p* = 0.08).

### sTREM2 may be more valuable in patients with disease course >12 months

**Figure 3:**
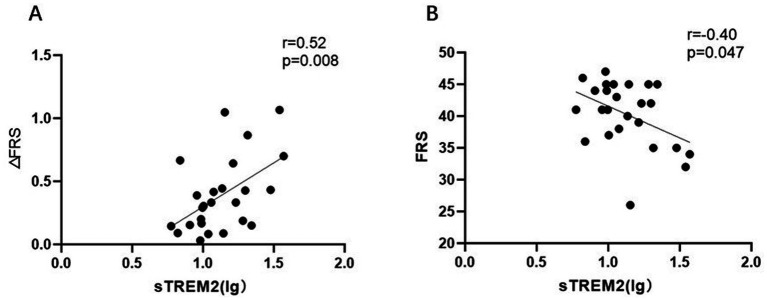
Subgroup analysis: ALS patients with a disease course >12 months. **(A)** Positive correlation between sTREM2 levels (lg) and the ΔFRS. **(B)** The negative correlation between sTREM2 levels (lg) and FRS.

Considering that the role of microglia may differ across different stages of the disease, we performed subgroup analysis. The ALS group was divided into subgroups according to the duration of disease (bounded by 12 months). When this correlation was estimated only among patients with a disease course >12 months, the sTREM2 (lg) was still associated with the UMN score (*r* = 0.50, *p* = 0.01), ΔFS (*r* = 0.52, *p* = 0.008) and serum NFL (lg) (*r* = 0.55, *p* = 0.004). In addition, there was a significant negative correlation (*r* = −0.40, *p* = 0.047) with ALSFRS-R (Figure 3). Nevertheless, no association was found in the subgroup with a disease duration <12 months. It seems to indicate that the role of sTREM2 in different pathological stages of the disease is not entirely consistent.

## Discussion

In this study, we found that CSF sTREM2 levels in patients with ALS were elevated and correlated with the UMN burden, which may provide some diagnostic support, especially for patients with insignificant upper motor neuron involvement. In addition, we found that patients with high sTREM2 levels had faster disease progression, which suggests a potential biomarker for disease monitoring in ALS patients.

sTREM2 may be a candidate indicator of upper motor unit involvement in ALS. The sTREM2 protein, which is expressed mainly in microglia of the central nervous system and is abundant in cerebrospinal fluid, has been evaluated as a biomarker of microglial activation (15), playing a key role in the signaling pathway that triggers microglial activation. A recent study revealed that activation of the pyroptosis pathway in ALS white matter microglia contributes to neuronal degeneration in the motor cortex and pyramidal tract, possibly by affecting axonal health (16).

In this study, the UMN score and MBS were used as indicators of the severity of upper motor neuron involvement. We observed that the sTREM2 level tended to increase in ALS patients with lower MBS signals and wider distributions. Iron deposition in the brain mainly occurs in malnourished microglia, and inflammatory changes and blood-brain barrier disorders play roles in iron homeostasis (17). Activated microglia can play a role in phagocytosis, swallowing excess iron in neurodegenerative diseases (18). In turn, iron overload impairs the motility of microglia and promotes aging and proinflammatory phenotypes (19). However, the direct relationship between sTREM2 and iron deposition has not been fully elucidated. Shi et al. (20) reported that CSF ferritin can act as an alternative biomarker of sTREM2-mediated microglial function in AD patients, playing an upstream role in inducing neuroinflammation. Previous studies have suggested that the “motor band sign” is related to the iron deposits shown at autopsy (21, 22). These degenerative iron accumulations are observed as hypointensities in the primary motor cortex on T2-weighted, T2*-weighted or susceptibility-weighted imaging (SWI), which are more predominant with UMN-ALS than with LMN-ALS (23, 24). Therefore, we included the MBS and speculated that there may also be a certain connection between the MBS and sTREM2.

sTREM2 may be a candidate indicator for disease progression in ALS patients. The sTREM2 may bind to pathological proteins and may affect signal communication with nearby cells (25), which raises the possibility that sTREM2 may be causally linked to disease progression. In this study, we found a close positive correlation between sTREM2 and ΔFS and NFL, indicating a link with the pathological phenomenon of axonal damage (26). Consistent with previous studies (27), no association was found with the FRS, but we did find an association in patients with an advanced disease course (>12 months). We speculate that the early increase in sTREM2 expression may reflect an initial immune response to the deposition of pathological aggregates, but may initiate destructive effects after a certain stage. It is likely that microglia adopt an M2-like neuroprotective state early in disease but transition to an M1-like toxic state as ALS progresses, and such transitions in the microglial activation state occur asynchronously (28). However, Rosén et al. (29) reported no difference in CSF sTREM2 levels among ALS patients, mimics, and controls. The reason for this difference may be due to differences in the research subjects and the detection methods used. In the study by Rosén et al. (29), 9 patients presented with manifestations of FTD, and 3 patients had a family history of ALS; our study subjects were patients with sporadic ALS. Because ALS is a highly heterogeneous disease, differences in inclusion criteria and disease stage may also affect outcomes. In addition, we used ELISA to analyze samples, whereas Rosén et al. (29) used the Meso Scale Discovery method.

Previously, microglial sTREM2 was proposed as a novel biomarker for other neurological inflammatory disorders (30–32), such as AD and MS. Recent studies have demonstrated an interaction between TDP-43 and TREM2 *in vitro* and *in vivo* as well as in ALS patient tissues by mass spectrometry and surface plasmon resonance analysis (33).

In this study, we found that CSF sTREM2 may also be a biomarker for disease monitoring in ALS patients.

This research, however, is subject to several limitations. First, the sample size is small, which can lead to sample bias or the ability to extend the results to the ALS population. Second, the lack of analysis of longitudinal patient data, including clinical data and sTREM2 levels, may limit any conclusions about progression. Third, the semiquantitative analysis of the MBS using the 3 Tesla MR system in this study may not be as accurate as the quantitative analysis (7 T QSM sequence). Therefore, the results should be replicated in large-sample, longitudinal data to analyze the effects of CSF sTREM2-associated inflammatory activity at different stages of the disease and to determine whether it is associated with disease progression.

## Conclusion

In conclusion, our results support that CSF sTREM2 may serve as a novel biomarker of disease monitoring for ALS, indicating upper motor neuron and axonal damage. Characterization of biomarkers of CSF sTREM2 and microglial activation will increase the understanding of potential neuroimmune mechanisms relevant to ALS.

## Data Availability

The original contributions presented in the study are included in the article/supplementary material, further inquiries can be directed to the corresponding authors.
